# Abrupt darkening under high dynamic range (HDR) luminance invokes facilitation for high-contrast targets and grouping by luminance similarity

**DOI:** 10.1167/jov.20.7.9

**Published:** 2020-07-14

**Authors:** Chou P. Hung, Chloe Callahan-Flintoft, Paul D. Fedele, Kim F. Fluitt, Onyekachi Odoemene, Anthony J. Walker, Andre V. Harrison, Barry D. Vaughan, Matthew S. Jaswa, Min Wei

**Affiliations:** Human Research and Engineering Directorate, CCDC Army Research Laboratory, U.S. Army, Aberdeen Proving Ground, MD, USA; DCS Corporation, Alexandria, VA, USA; Computational and Information Sciences Directorate, CCDC Army Research Laboratory, U.S. Army, Adelphi, MD, USA

**Keywords:** luminance normalization, orientation discrimination, target recognition, machine vision, computer vision

## Abstract

When scanning across a scene, luminance can vary by up to 100,000-to-1 (high dynamic range, HDR), requiring multiple normalizing mechanisms spanning from the retina to the cortex to support visual acuity and recognition. Vision models based on standard dynamic range (SDR) luminance contrast ratios below 100-to-1 have limited ability to generalize to real-world scenes with HDR luminance. To characterize how orientation and luminance are linked in brain mechanisms for luminance normalization, we measured orientation discrimination of Gabor targets under HDR luminance dynamics. We report a novel phenomenon, that abrupt 10- to 100-fold darkening engages contextual facilitation, distorting the apparent orientation of a high-contrast central target. Surprisingly, facilitation was influenced by grouping by luminance similarity, as well as by the degree of luminance variability in the surround. These results challenge vision models based solely on activity normalization and raise new questions that will lead to models that perform better in real-world scenes.

## Introduction

Vision is an inherently ambiguous process of estimating and predicting the true three-dimensional (3D) world from a two-dimensional retinal image, and the apparent ease of vision is belied by the fact that nearly half of the brain is devoted to visual processing. Understanding luminance normalization, how the brain untangles myriad factors to estimate reflectance and shape, is important both for modeling the brain's computational principles and for building resilient machine sensing for real-world environments.

**Figure 1.**
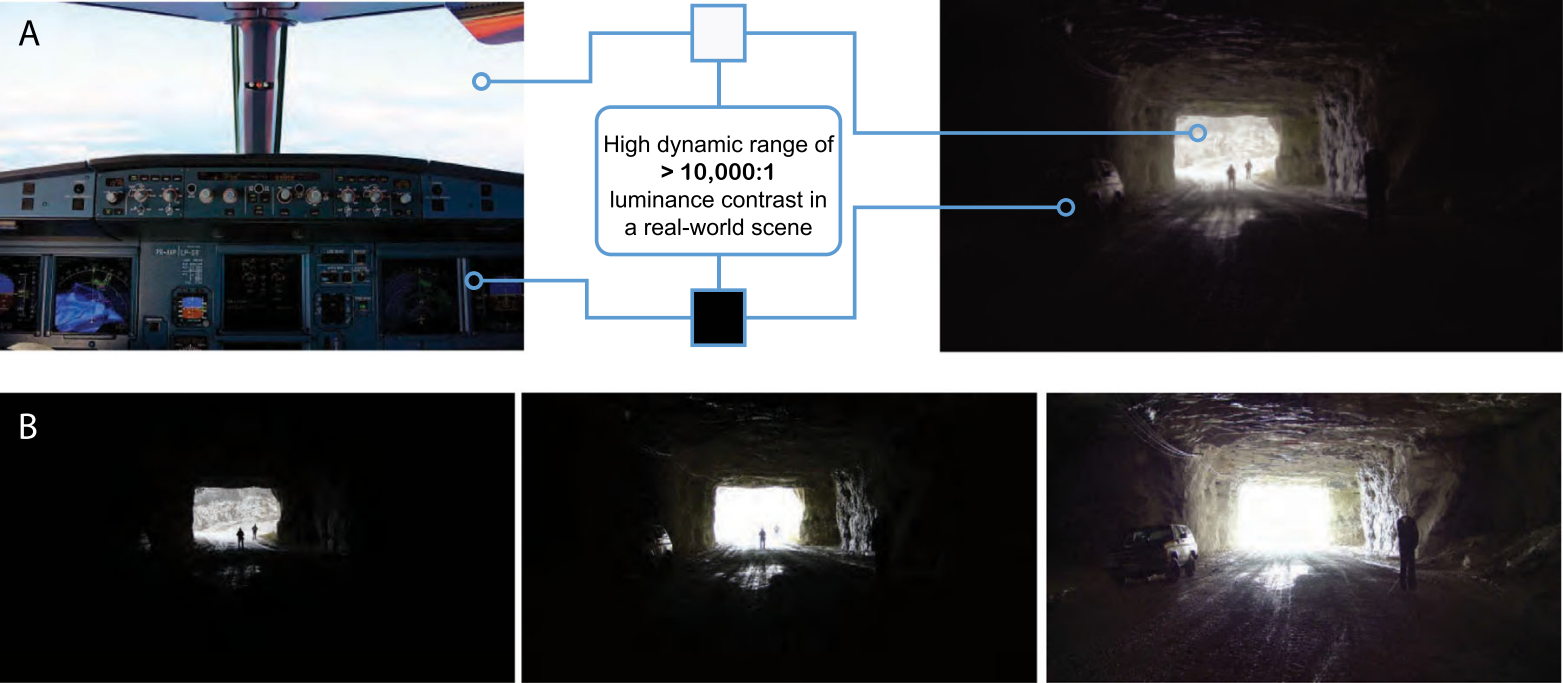
(A) Two examples of high dynamic range (HDR) luminance in naturalistic scenes: cockpit view (left) and view of a cave opening (right). These are examples of commonly encountered environments where combinations of indoor and outdoor luminance can exceed a 10,000-to-1 maximum-to-minimum luminance ratio. The scene at the right is a blended image across multiple exposures, illustrating our ability to see multiple targets (three uniforms and one car) across vast luminance differences in the same view. Actual views of these scenes appear even more vivid because of the brain's luminance normalization processes. (B) Standard dynamic range (SDR) 8-bit cameras are highly dependent on exposure, and machine vision systems that are highly dependent on texture patterns can easily miss targets due to under- or over-exposure. Exposure examples are at 10× increments.

Depending on the context of a visual scene, almost any luminance can appear as any shade of gray. This is because the luminance of the brightest and darkest areas of a scene can vary by a factor of up to 100,000-to-1 (Figure 1), whereas the surface reflectance information that is useful for estimating object shape and identity typically varies by a factor of only 20-to-1 (i.e., 4%–80% of the light illuminating the surface) (Gilchrist et al., 1999). Even slight (<0.5%) changes in illumination, due to variations in atmospheric haze or sun position, for example, can produce large (50%) average changes in luminance in a natural scene (e.g., due to cast shadows, foliage, or anisotropic reflectance effects such as microshadows) (Foster & Amano, 2019; Moore, 2010). Large dynamic changes, such as from flare effects and wind blowing on the leaves in a forest canopy (Marathe et al., 2017), can disrupt navigation and targeting algorithms that are overly reliant on texture patterns and on static luminance and illumination to compute optic flow. For autonomous ground vehicles, navigation is problematic because luminance normalization is not yet solved for recognizing distant targets (e.g., potholes, buried explosives) under multiple layers of optic flow, especially at high speeds and under degraded and novel conditions. This poses serious longstanding challenges to the real-world credibility and acceptability of autonomous maneuvering and targeting, with potentially catastrophic consequences (e.g., from false-positive or false-negative misclassifications).

For efficient and resilient recognition behavior, the human visual system has many automatic mechanisms to estimate reflectance and 3D shape, as demonstrated by brightness illusions (Adelson, 2000; Bach, 2019; Motoyoshi, Nishida, Sharan, & Adelson, 2007). Our understanding of these mechanisms, as quantified by models of normalization and visual salience, is limited because it is based on studies using displays with standard dynamic range (SDR) luminance (100-to-1 luminance ratio between the brightest and darkest pixels). Recent reports show that key theories such as Wallach's ratio rule, which states that the apparent lightness of a surface depends on the ratio of its luminance to the background, break down at high dynamic range (HDR) luminance (Allred, Radonjic, Gilchrist, & Brainard, 2012; Radonjic Hung, & Roe, 2011), in agreement with previously demonstrated deviations from the rule at SDR luminance such as disk annulus figures (Rudd, 2017). To investigate and understand these mechanisms and thereby improve the real-world performance of vision models, it is necessary to investigate perception under HDR luminance.

To achieve illumination-resilient recognition, biological visual systems gradually transform the retinal image from a luminance-based representation to reflectance-approximated, feature-based representations (Janssen, Vogels, Liu, & Orban, 2001; Roe et al., 2012; Wachtler, Sejnowski, & Albright, 2003; Zhou, Friedman, & Von Der Heydt, 2000). Evidence from anatomy, electrophysiology, and behavior shows that this process is supported by a hierarchy of over 30 visual cortical areas that combine recurrent and lateral processes with feedback mechanisms for contextual normalization (Ramsden et al., 2001; Tanigawa, Wang, Q., & Fujita, 2005). The brain areas that support normalization are highly specialized and include specific domains for luminance and color processing within multiple visual cortical areas, including the primary visual cortex (area V1, the first brain area to receive input from the retina and thalamus) (Conway, Moeller, & Tsao, 2007; Kremkow, Jin, Wang, & Alonso, 2016; Lim, Wang, Xiao, Hu, & Felleman, 2009; Roe et al., 2012; Schroeder, Mehta, & Givre, 1998; Wang, Xiao, & Felleman, 2007). The circuitry underlying these processes is less understood, but there is evidence for tight coupling between processes for luminance/color and processes for form perception, including feedback circuitry from the last stage of the visual form pathway to V1 (Clavagnier, Falchier, & Kennedy, 2004; Hung, Ramsden, & Roe, 2007; Hung, Ramsden, & Roe, 2010; Ts'o, Roe, & Gilbert, 2001). Rather than simply remapping the color histogram or normalizing an image for nearby luminance, automatic mechanisms are thought to depend on factors such as co-orientation, co-linearity, co-circularity, co-planarity, junctions, feature grouping, and transparency issues, such as smoke and rain (Adelson, 2000; Anderson, 1997; Li, Song, Xu, Hu, Roe, & Li, 2019; Zemach & Rudd, 2007; Zucker, David, Dobbins, & Iverson, 1988). Biologically driven models are increasingly capable of explaining visual illusions (Blakeslee & McCourt, 2004; Li, 2011) and predicting gaze patterns based on saliency (Kümmerer, Wallis, Gatys, & Bethge, 2017; Wang, Shen, Xie, Cheng, Ling, & Borji, 2019), but they are data-limited to SDR images and require HDR experimentation to extend their generalizability to real-world vision.

We investigated contextual mechanisms for luminance normalization by testing for interactions between orientation and HDR luminance processing. Previous reports of contextual orientation effects found that flankers drive a facilitating response (making a co-oriented target easier to detect) if the target is low contrast, and this observation was initially attributed to horizontal fibers linking V1 neurons that prefer the same orientation (Ts'o, Gilbert, & Wiesel, 1986). However, at higher contrast, a co-oriented target becomes more difficult to detect than an orthogonal target, an effect that is consistent with suppression of the target visibility or assimilation of the target to surrounding co-oriented patterns, possibly due to feedback from higher pattern-sensitive cortical areas. Both phenomena have also been attributed to the balance of local recurrent excitatory and inhibitory mechanisms in V1, but they have thus far only been investigated for static luminance displays and uniform patch luminance (Chen & Tyler, 2001; Chen & Tyler, 2002; Chen, Kasamatsu, Polat, & Norcia, 2001; Li, 1998; Li, 2011; Polat & Sagi, 1993; Polat & Sagi, 2006; Polat, Mizobe, Pettet, Kasamatsu, & Norcia, 1998).

We reasoned that in naturalistic vision, our gazes often shift across regions with large (100×) differences in luminance. How does the visual system normalize quickly, perhaps even predictively (perisaccade), across large luminance changes, and can we discover such a normalizing mechanism (e.g., linking luminance and form vision) by observing how shape perception is altered when shifting one's gaze from light to dark areas of the visual scene during visual search? Would strong darkening lead to contextual facilitation, even for high-contrast targets, or suppression/assimilation? Also, would the contextual effect be driven by the brightest flankers, consistent with models of recurrent excitation/inhibition and divisive or subtractive normalization, or would the effects be driven by the flankers that are most similar in luminance to the target, consistent with assimilation or feedback from higher brain areas? To answer these questions, we took the novel approach of combining (1) a luminance transition of 1×, 10×, or 100×; (2) the 5-by-5 array pattern of recent HDR luminance studies (Radonjic, Allred, Gilchrist, & Brainard, 2011); and (3) oriented lines (flanker Gabors) that surround the target and affect target visibility (classic contextual orientation effects discovered with static SDR displays; Chen & Tyler, 2002). We then measured how these contextual HDR luminance manipulations affect orientation discrimination of a central target.

## Methods

### Subjects

Nine subjects (six male) 18 to 70 years old participated in all experiments. Potential subjects were excluded if they self-reported that they, their parents, or their siblings had photosensitive epilepsy; if they previously had head trauma or other disorders thought to be associated with excitatory/inhibitory balance (epilepsy, schizophrenia, autism, depression, attention deficit hyperactivity disorder) (Jiang et al., 2013); if they had atypical brain development; or if they had used mind-altering drugs in the past week. Potential subjects were also screened via the Canadian Longitudinal Study on Aging–Epilepsy Algorithm (Keezer, Pelletier, Stechysin, Veilleux, Jetté, & Wolfson, 2014). Subjects were de-identified via random letters. All experiments were conducted at the Army Research Laboratory at Aberdeen Proving Ground, MD, according to a protocol approved by the Army's Human Research Protection Program.

### Vision screening

Prior to beginning experimental tasks, subjects were screened for normal or corrected-to-normal (at least 20/40) visual acuity and normal color vision via a Titmus i500 Vision Screener (Honeywell, Charlotte, NC). Visual acuity under dynamic luminance was also assessed via a custom logMAR acuity task (Hung et al., 2019; Hung, Callahan-Flintoft, et al., 2020).

### HDR display and eye tracking

All images were projected from a DLA-RS600U 4K Reference Projector (JVC, Kanagawa, Japan) and displayed biocularly on an HD projection screen (Hung et al., 2019; Hung, Callahan-Flintoft, et al., 2020). Images spanned 1920 × 1080 pixels in resolution (48.7 cm × 27.3 cm, width × height) and were observed from a chin-rest-stabilized viewing distance of 78 cm, thus spanning a 34.7° × 19.9° viewing angle with pixel size 0.0181° × 0.0184°. Gaze and pupil size were tracked monocularly via an infrared eye tracker (EyeLink 1000 Plus; SR Research, Ltd., Kanata, ON, Canada), synchronized via Lab Streaming Layer software (Swartz Center for Computational Neuroscience, University of California San Diego, San Diego, CA) (Kothe & Makeig, 2013). To maintain a constant peak luminance in the visual field, all tasks included static 400 cd/m^2^ light anchors (Gilchrist et al., 1999), 1° × 1° in size, at the four corners of the screen.

Images were displayed at 60 Hz and pseudo 11 bits (10.7 bits; 11 bits red and 11 bits green but only 10 bits blue, because all of the color information needs to fit into 32 bits) grayscale precision via a framebuffer procedure using Psychtoolbox 3.0 (Kleiner, Brainard, & Pelli, 2007) for GNU/Linux X11 software running under MATLAB 64-bit version 2016b (MathWorks, Natick, MA) on Ubuntu 16.04 (Canonical Ltd., London, UK) and an AMD FirePro W8100 graphics card (Advanced Micro Devices, Santa Clara, CA). We used the Psychtoolbox command PsychImaging(‘AddTask’, ‘General’, ‘EnableNative11BitFramebuffer’) to disable and bypass the hardware's gamma color lookup table and switch the framebuffer into 11-bpc mode, verified by spectrophotometer (PR-745; Photo Research, Los Angeles, CA). We then applied a 75-point log-linear gamma correction, and the resulting gray levels spanned a range of 636.4 cd/m^2^ (*u*, *v* = 0.1953, 0.3199; *x*, *y* = 0.3200, 0.3494; 6037K) to 0.006055 cd/m^2^ at regular log-linear steps, for a maximum contrast ratio of over 100,000-to-1 in a single image (static projector iris) (Hung, Callahan-Flintoft, et al., 2020). This range spans mesopic vision (0.001–3 cd/m^2^, when both cones and rods are required to support vision) to the lower end of photopic vision (10–10^8^ cd/m^2^), consistent with seeing in mixed indoor/outdoor environments and in twilight (e.g., nighttime street, outdoor lighting, aviation lighting).

### HDR target discrimination task

We developed a two-alternative forced-choice HDR target discrimination task comprised of five experimental blocks to test for interactions between luminance dynamics and orientation. In three blocks, we tested different luminances of an adapting blank screen, including 4 cd/m^2^ (HDR gray, no change), 40 cd/m^2^ (HDR light gray), and 400 cd/m^2^ (HDR white), followed by an HDR target and flanker array. We tested two additional control blocks, one consisting of a 40-cd/m^2^ light gray adapting blank followed by a narrower luminance range SDR target and flanker array (SDR block), and the other a dynamic variant of classic flanker stimuli with a 40-cd/m^2^ light gray adapting blank followed by a 4-cd/m^2^ uniform background and uniform flanker orientation (uniform block). Blocks consisted of 400 trials each and were presented in the following order: uniform, HDR light gray, SDR light gray, HDR gray, and HDR white.

**Figure 2.**
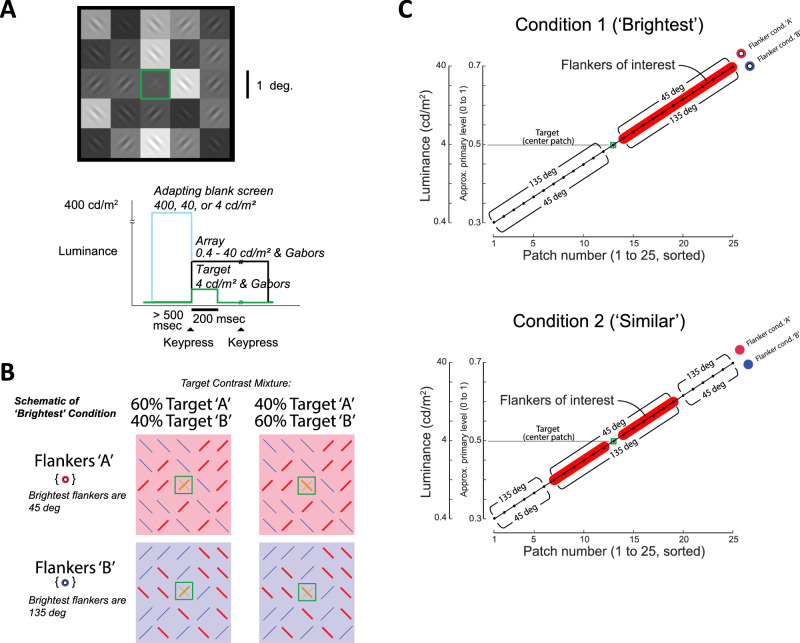
Stimulus and task design. (A, top) Example stimulus with target contrast mixture of 60% A (45°) and 40% B (135°). (A, bottom) Time course of adapting blank screen, keypresses, and array and target presentations. (B) Schematic of example trial types for the brightest condition for the example stimulus. In this condition, the brightest flankers are the flankers of interest (red lines) and are oriented at either 45° (flanker condition A) or 135° (flanker condition B). Only two of five possible target contrast mixtures (A/B) are shown (30%/70%, 40%/60%, 50%/50%, 60%/40%, 70%/30%). For the similar condition (not shown), the flankers of interest would be flankers that were most similar in luminance to the target. The brightest and similar condition trials were pseudorandomly interleaved within a block. (C) Orientation/luminance relationship of 24 flankers for flanker condition A (angle labels above diagonal) and flanker condition B (below diagonal) in the brightest (top) and similar (bottom) conditions. Red highlights indicate the flankers of interest for each condition, and the green square indicates the target. In each of the HDR luminance blocks, flanker patches span 40 to 0.4 cd/m^2^ (133–0.12 cd/m^2^ full range including Gabors). This range is reduced to 12.6 to 1.26 cd/m^2^ (42–0.378 cd/m^2^ full range) for the SDR block.

The target and flanker array consisted of 45° and 135° Gabors, 4 cycles/degree and 1° full width at half maximum Gaussian envelope, cropped to 1° × 1° and presented on a 5 × 5 array of 1° × 1° luminance patches (Figure 2A, top). The spatial frequency of the Gabors is consistent with the stimulus preferences of single neurons in primary visual cortex with receptive fields at 3° eccentricity (Chu, Chien, & Hung, 2014).

Across all blocks, the target was a contrast mixture of two Gabors at 45° (A, tilted right) and 135° (B, tilted left), presented at the central patch at a fixed mean luminance of 4 cd/m^2^, and subjects indicated via keypress the orientation of the stronger target Gabor. At full contrast (100%), the pixels of each Gabor spanned 3.3× to 0.3× its patch luminance (less than the requested range of 10× to 0.1× patch luminance, due to projector light scatter) (Hung, Callahan-Flintoft, et al., 2020; Hung, Larkin, et al., 2020). The target was shown at one of the five possible A:B contrast mixtures of 70%:30%, 60%:40%, 50%:50%, 40%:60%, or 30%:70%. These contrast mixtures were logarithmically applied to each full-contrast Gabor, such that 50%:50% means that the brightest and darkest pixels of both Gabors are 1.8× and 0.55× the target patch luminance. The five target mixtures were tested in all blocks and conditions. All Gabor patterns were well above the threshold contrast visibility for normal vision. At the minimum average luminance of 0.4 cd/m^2^, in a field of view 1° by 1°, and at a modulation frequency of 4 cycles/degree, Barten (Barten, 2003) reported a minimum visible contrast ratio of 1.03; for a field of view 0.5° by 0.5°, the minimum visible contrast ratio increased only to 1.06.

The flanker patches evenly log-linearly spanned a luminance range from 40 to 0.4 cd/m^2^ (10× to 0.1× the target patch luminance) for HDR blocks and from 12.6 to 1.26 cd/m^2^ (3.16× to 0.316× the target patch luminance) for the SDR block, and they were uniformly 4 cd/m^2^ for the uniform block. The pixels of each Gabor spanned a range from 3.33× to 0.3× its patch luminance, resulting in a peak contrast including Gabors of 1111-to-1 (133–0.12 cd/m^2^) for the HDR blocks, 111-to-1 (42–0.378 cd/m^2^) for the SDR block, and 11-to-1 (13.3–1.2 cd/m^2^) for the uniform block.

We tested two orthogonalized conditions in which we assigned flankers of interest to different orientation/luminance combinations to determine whether the behavioral biases were driven by the brightest flankers or the flankers that were most similar to the target in luminance. Each 5 × 5 grid consisted of an inner ring of eight patches and an outer ring of 12 patches. The flankers of interest were balanced within each ring, such that they had the same number of co-oriented and orthogonal flankers, and their locations were spatially balanced in the horizontal and vertical directions to avoid highly asymmetric patterns. In the brightest condition, the flankers of interest were the 12 brightest patches. In the similar luminance condition, the flankers of interest were the 12 patches most similar in luminance to the target patch. For both conditions, we defined flanker condition A as the case in which the Gabor flankers of interest were oriented 45° and the remaining flankers were 135°, and vice versa for flanker condition B. The brightest and similar trials were pseudorandomly interleaved within a block.

At the start of every trial, a black fixation cross appeared at the center of the adapting blank screen. After a minimum blank of 500 ms, pressing the spacebar initiated a sequence that began with the offset of the blank screen and fixation cross, replaced by a black screen (0.006055 cd/m^2^) (Figure 2A, bottom). The 5 × 5 flanker array including the 4 cd/m^2^ target patch appeared 50 ms later and then the target Gabors 17 ms after that. The target Gabors remained on the screen for 250 ms and then offset, leaving the flankers on the screen until the participant hit the left or right arrow key to report the orientation of the stronger target Gabor. After a keypress, the blank screen reappeared after 500 ms, ending the trial.

### Behavioral analysis

Behavioral choices were fitted with a psychometric function based on a cumulative Gaussian distribution, using Psignifit software running in Python (Fründ, Haenel, & Wichmann, 2011). Psignifit estimates a free guessing and lapse rate parameter (options.expType = ‘YesNo’) to fit the behavior via maximum likelihood. Compared to classic logarithmic fitting tools, Psignifit is thought to provide better confidence intervals by avoiding two critical assumptions of stability and binomial distribution to account for factors such as learning, fatigue, and fluctuations of attention.

To analyze the significance of the flanker-induced bias for each condition, we defined a significant behavioral effect as cases where the 5% to 95% confidence interval at the 50%-choose-A threshold of each curve did not cross the other curve for the two curves from flanker condition A and flanker condition B. We analyzed this bias separately for the brightest and similar conditions and for each of the blocks. For population analysis, we defined each subject's bias as the difference in the target mixtures of the two curves at the 50%-choose-A threshold. We then applied a one-sample, two-tailed *t*-test to examine the significance of this bias for each condition, versus a null hypothesis of zero bias across the population. We also compared these biases across the brightest and similar conditions via two-tailed, paired *t*-test. Significance tests were not corrected for multiple comparison.

## Results

### Study design

Previous reports of contextual effects on target orientation discrimination were based on uniform flanker orientations against a uniform, static background luminance. In previous reports, the visibility of a low-contrast target Gabor was enhanced by surrounding co-aligned flankers (facilitation), but the visibility of a moderate-contrast target was reduced (suppression or assimilation), and we confirmed this during pilot testing. In electroencephalogram recordings, flanker co-linearity also produced an increased midline occipital positive polarity between 80 to 140 ms after stimulus onset, consistent with a mechanism in area V1 (Khoe, Freeman, Woldorff, & Mangun, 2004; Polat & Norcia, 1996). This dichotomy of contextual facilitation versus suppression to static SDR stimuli has formed the basis of computational models of V1, based on a balance of recurrent excitation and inhibition (Chen & Tyler, 2002; Li, 2011).

To understand and model the contextual mechanisms of luminance normalization under real-world luminance dynamics, we introduced two changes to the classic flanker task: (1) a preceding adapting blank background to mimic the luminance change across gaze shifts, and (2) a 5 × 5 array of luminance patches spanning a 10- or 100-fold difference in luminance to mimic the conjunction of form and luminance in naturalistic scenes. This combination of adapting blanks, patches, and Gabors resulted in a total luminance range of 3333-to-1: 400 cd/m^2^ for the brightest adapting blank versus 0.12 cd/m^2^ for the darkest Gabor pixel.

We tested this combination via a two-alternative forced-choice task in which subjects reported the orientation of the stronger of two Gabor targets shown at the center of the 5 × 5 array (Figure 2A). By fitting the behavioral responses across target contrast mixtures with a psychometric function, we were able to determine whether the flankers induced a facilitatory or suppressive/assimilation effect under real-world luminance dynamics. Additionally, by manipulating the conjunction of patch luminance and patch orientation via two orthogonalized conditions, we were able to test alternative hypotheses about normalization mechanisms that predict whether the brightest flankers or the flankers having a luminance most similar to the target would have stronger effect.

To examine how contextual luminance and orientation combine to affect target discrimination, we manipulated the conjunction of luminance and orientation across the 5 × 5 array of patches. Figure 2B illustrates schematic examples of such stimuli for the brightest condition in which the flankers of interest (indicated by red lines) are at the brightest patches. In the upper left example of Figure 2B, corresponding to the stimulus example in Figure 2A, the target mixture is 60%/40% and the flanker condition is A, so both the target and the flankers of interest are tilted to the right (45°). The comparison condition with the identical target mixture is flanker condition B (lower left), in which the flankers of interest are tilted to the left (135°).

Figure 2C (top) illustrates this comparison of orientations for flanker condition A (red open circle, angles above diagonal) versus flanker condition B (blue open circle, angles below diagonal) for the brightest condition, sorted by patch luminance. The red highlights indicate the flankers of interest (the brightest patches in this brightest condition).

If the behavioral bias were driven by the brightest flankers, we would expect to see a difference in the target report between flanker condition A and flanker condition B in the brightest condition. The same direction of bias should be present across a range of target mixtures, including when the target mixture is 40%/60% (upper and lower right examples in Figure 2B).

Conversely, if the behavioral bias were driven by the flankers that are most similar in luminance to the target, the effect would be stronger in the similar condition, in which the flankers of interest are at the 12 patches most similar to the target in luminance (Figure 2C, bottom). The effect should be abolished in the brightest condition, when flankers near the target luminance are both co-oriented and orthogonal.

### Individual subject examples

**Figure 3.**
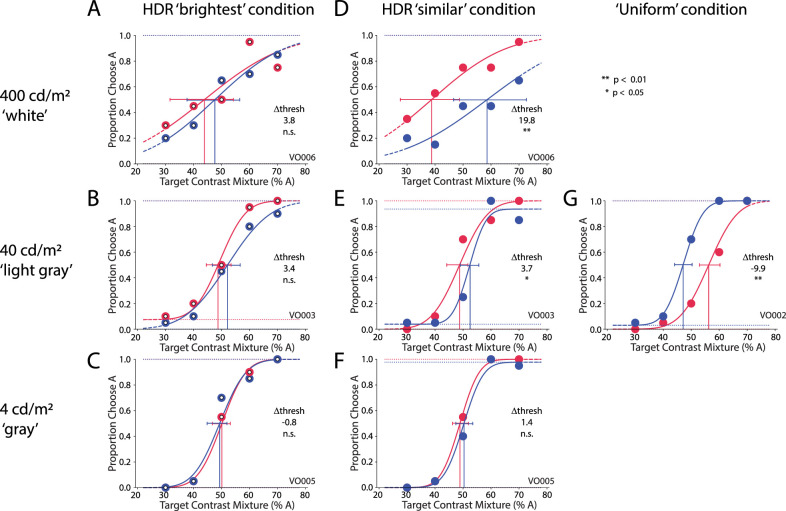
Subject VO's perceptual report of the target Gabor orientation, broken out by block and condition. Adapting blank screens were 400 cd/m^2^ white (A, D), 40 cd/m^2^ light gray (B, E, G), or 4 cd/m^2^ gray (C, F). Red curves and data show perceptual reports when the flankers of interest were tilted to the right (45°, flanker condition A); blue curves and data show perceptual reports when the flanker of interest were tilted to the left (135°, flanker condition B). Threshold bias (Δ-thresh) is the difference in target contrast mixture at a 0.5 proportion of choosing A. Positive threshold bias (red curve left of blue curve) indicates that the subject tended to choose A when the flankers of interest were also A. (G) In the uniform block, when all flankers were the same orientation and 4 cd/m^2^ luminance, subject VO's bias was away from the flanker orientation. Error bars show 5% to 95% confidence intervals from 200 trials per figure panel, based on Psignifit.

Figure 3 shows an example of the behavioral results for one subject, VO. As expected, the subject tended to choose A (45° target) when the target contrast mixture was 60%/40% or 70%/30% and B (135° target) when the target contrast mixture was 30%/70% or 40%/60%, indicating that the subject was generally able to correctly perceive the dominant orientation of the target. For the brightest condition (Figures 3A–3C), the psychometric function did not significantly differ between flanker conditions A (red circles) and B (blue circles) across all three adapting blank luminance levels 400, 40, and 4 cd/m^2^, indicating that the brightest flankers failed to bias behavioral choice.

Conversely, the flankers induced a significant bias on target choice behavior in the similar condition when the flankers of interest were at the 12 patches most similar in luminance to the target. The effect was facilitatory; there was a bias in the subject's response toward the orientation of the flankers of interest, as indicated by the leftward shift of the red curve (increased likelihood of choosing A 45° when the flankers of interest were A 45°) and the rightward shift of the blue curve (increased likelihood of choosing B when the flankers of interest were B 135°) in Figures 3D and 3E. This flanker-induced bias was significant for the two brightest adapting blank screen luminances, 400 cd/m^2^, and 40 cd/m^2^ (threshold bias, the difference in target contrast mixture for flanker condition A vs. B at the 50%-choose-A threshold, was 19.8 at 400 cd/m^2^ and was 3.7 at 40 cd/m^2^; *p* < 0.01 and *p* < 0.05, respectively), but it was not significant when the luminance at the target patch was unchanged at 4 cd/m^2^ adapting blank (Figure 3F). In pilot testing, we also observed no significant bias for darker adapting blank screens of 0.04 and 0.4 cd/m^2^. In the uniform block (Figure 3G), when a 40-cd/m^2^ adapting blank was followed by all flankers having the same orientation and the same 4-cd/m^2^ patch luminance, subject VO's report was significantly biased away from the flanker orientation, consistent with suppression or assimilation (threshold bias = –9.9; *p* < 0.01). In pilot tests without the adapting blank screen, we also observed the same bias across all subjects, consistent with previous reports of suppression at higher target contrasts.

**Figure 4.**
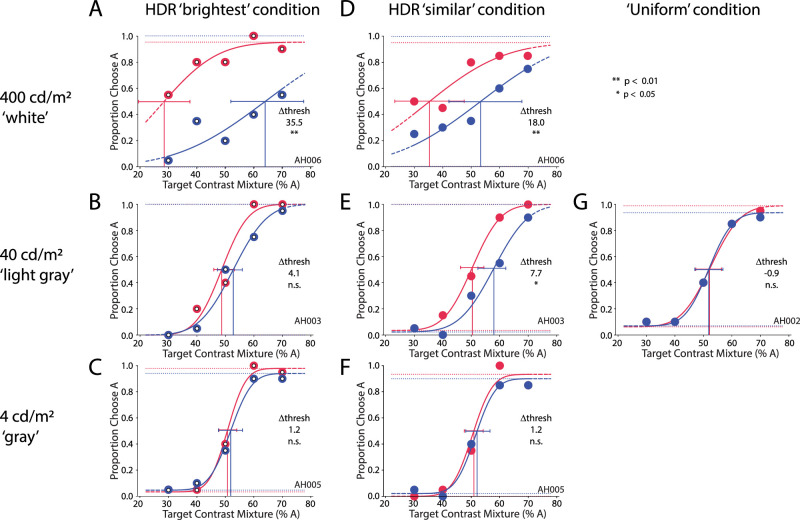
Subject AH's perceptual report of the target Gabor orientation, broken out by block and condition as in Figure 3.

Another subject, AH, also showed a flanker-induced bias toward facilitation, but it was significant in both the brightest and similar conditions for the 400-cd/m^2^ HDR white adapting blank (threshold biases = 35.5 and 18.0, respectively; *p* < 0.01 in both cases) (Figures 4A and 4D). The strength of this bias, a difference of 35.5% in the brightest condition, is illustrated by the fact that, even when the target mixture was 70% B, the subject chose A 60% of the time under flanker condition A, and the subject chose B 97% of the time under flanker condition B. Conversely, when the target was 70% A, the subject chose A 90% of the time under flanker condition A, but chose B 45% of the time (chose A 55% of the time) under flanker condition B. As with subject VO, subject AH also showed a facilitatory bias for the similar condition in the 40-cd/m^2^ HDR light-gray block (Figure 4E) and no significant bias in the remaining HDR blocks (Figures 4B, 4C, 4F). However, unlike subject VO, subject AH showed no significant bias in the uniform condition.

### All subjects

**Figure 5.**
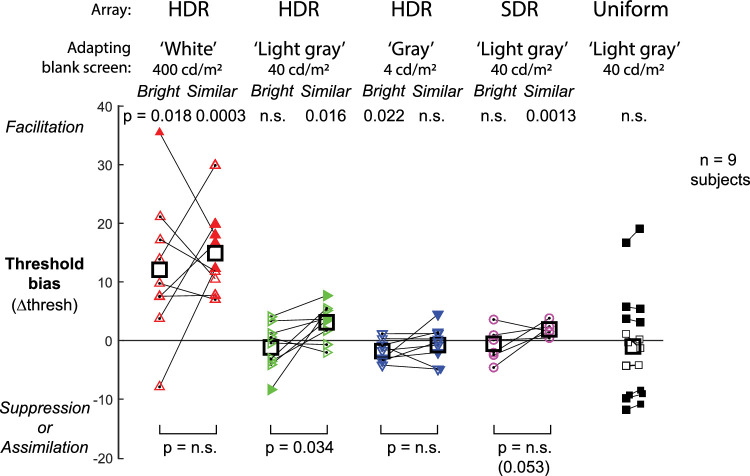
Results for all subjects. Filled symbols show cases where the subject's perceptual report depended significantly on the orientation of the flankers of interest, consistent with facilitation (positive bias) or suppression/assimilation (negative bias). Thick boxes show population means, and lines connect the brightest and similar conditions for the same subject. The upper statistics show the significance of each condition for all subjects (*n* = 9; 200 trials per condition per subject; unpaired *t*-test), and the lower statistics show the significance of the pairwise comparison across the brightest and similar conditions of the same block. For the uniform block, connected squares in the left and right columns show the results of split trials, 200 trials per square, indicating the precision of the measurement for each subject.

Across subjects, there was a strong flanker-induced bias toward facilitation when the adapting screen was much brighter than the Gabor display; that is, there was a strong bias to report targets as co-oriented with the flankers of interest (Figure 5). At the brightest adapting blank of 400 cd/m^2^ (100× brighter than the target patch, HDR white), this flanker-induced facilitation was strong and significant for both the brightest and similar conditions (threshold biases = 12.0 ± 12.1 and 14.8 ± 7.2; *p* = 0.018 and *p* = 0.0003, respectively), and there was no significant difference between these two conditions, indicating that both the brightest flankers and the flankers similar to the target in luminance contributed to the facilitation.

The effect depended on the magnitude of the luminance change. When the adapting blank was only 10× brighter than the target patch (HDR light gray), the flanker-induced facilitation was significant for the similar condition (threshold bias = 3.1 ± 3.1; *p* = 0.016), but this bias was abolished in the brightest condition, indicating that it was not driven by the brightest flankers (–1.2 ± 4.0; *p* = not significant). This difference between similar and brightest conditions was significant (*p* = 0.034, two-tailed, paired *t*-test).

When the adapting blank was the same luminance as the target patch (HDR gray), there was a weak but significant bias toward suppression in the brightest condition (–1.7 ± 1.8; *p* = 0.022), consistent with previous reports of suppression to high-contrast targets at static luminance. This bias was abolished in the similar condition (–0.06 ± 3.0; *p* = not significant), but the difference between conditions was not significant.

Could these effects have been observed with an SDR display? We tested an SDR light-gray condition in which the array patch luminances spanned only 12.6 to 1.26 cd/m^2^ (3.16× to 0.316× the target patch luminance, a 10× range) versus 40 to 0.4 cd/m^2^ (10× to 0.1× the target patch luminance, a 100× range) in the HDR array. As with the HDR light-gray condition, there was a significant flanker-induced facilitation in the similar condition (1.9 ± 1.0; *p* = 0.0013), but this effect was abolished in the brightest condition (–0.5 ± 2.5; *p* = not significant). The difference between these two conditions approached significance (*p* = 0.053). Thus, the effect was much weaker under SDR conditions but was consistent with the results under HDR conditions.

Across subjects, the uniform condition resulted in a wide variation of individual biases (–1.0 ± 9.4), ranging from significant facilitation (18.0; *p* < 0.01) to significant suppression (–11.8; *p* < 0.01). The bias was not due to noise in the measurement, as randomly splitting the trials resulted in almost no change to individual biases (Figure 5, pairs of connected squares). The direction of this individual bias did not appear to be related to the magnitude of facilitation in the HDR and SDR conditions. The wide range of individual biases under the uniform condition contrasts with the narrower range of biases in the HDR conditions, especially the SDR light-gray similar condition (1.9 ± 1.0). It also contrasts with previous reports (and our pilot results) of target suppression to static uniform flankers, indicating that our addition of the adapting light-gray blank substantially altered the conditions to produce unexpected behavior. We speculate that the blank, although only 10× brighter than the target patch, may increase ambiguity and enable the emergence of strong priors, based on the subjects’ false expectation of a relationship (match or mismatch) between the target and flankers.

### Effect of the degree of flanker patch luminance variability

In the lightness constancy literature, it has been suggested that structural complexity in the surrounding framework (articulation), including luminance variability, increases the degree of veridical lightness judgment and anchoring to that framework (Gilchrist et al., 1999; Zemach & Rudd, 2007). Although our subjects’ task was orientation discrimination, not lightness judgment, the facilitation observed in the similar condition suggests that orientation discrimination is influenced by grouping by luminance similarity. To what extent is this grouping-related facilitation effect titrated by the degree of luminance variability in the flankers, for the same level of abrupt darkening? Conversely, is it stronger for flankers that are identically the same luminance rather than merely similar?

**Figure 6.**
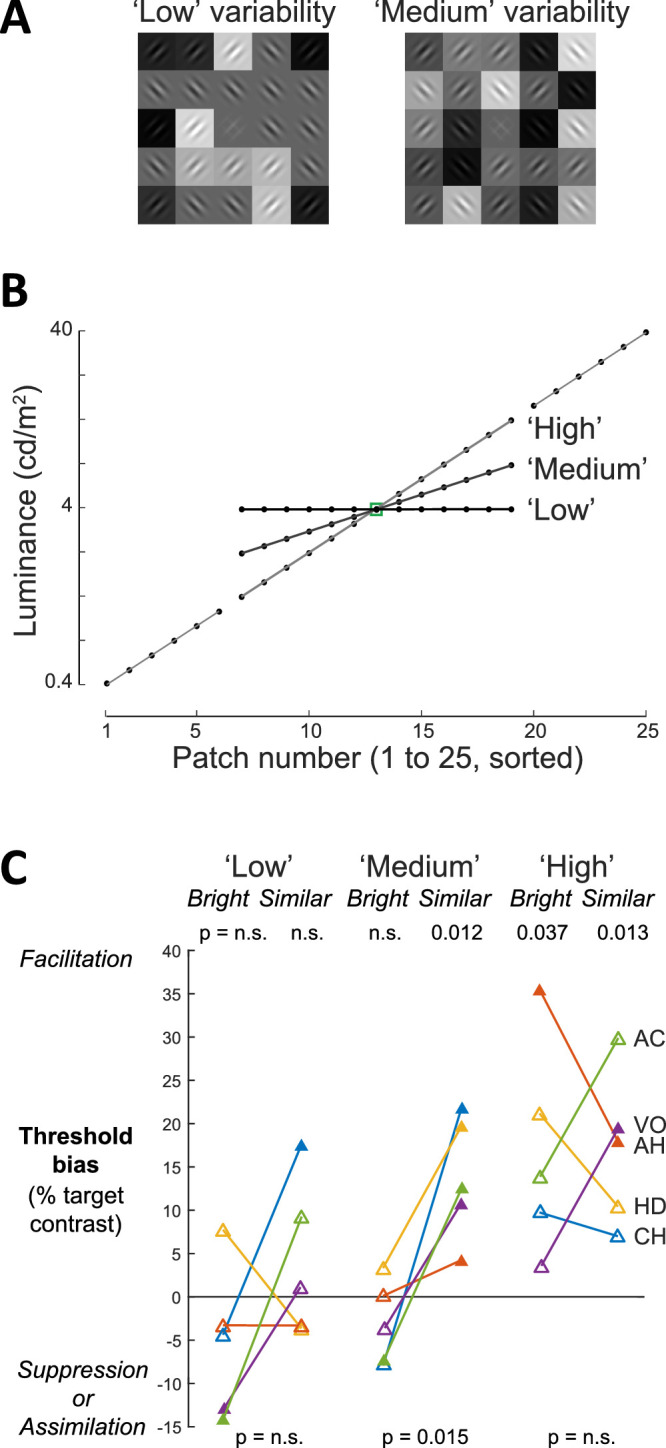
Effect of degree of flanker patch luminance variability. (A) Variants of the HDR white block in which the 12 flankers most similar in luminance to the target were either the same luminance as the target (low luminance variability, slope 0) or at half the original luminance range (medium variability, slope 0.5). Compare with the original luminances (high variability, slope 1, in Figure 2A). The remaining 12 flankers were identical across the three blocks. (B) Patch luminances for low, medium, and high luminance variability, identical for both similar and brightest conditions. (C) Threshold bias effects of similar versus brightest conditions for blocks at low, medium, and high luminance variability. Filled triangles indicate significant effects at *p* < 0.05 based on Psignifit of 200 trials per brightest or similar condition per block per subject; open triangles are non-significant. Upper and lower statistics are based on unpaired *t*-tests of individual conditions (200 trials) and paired *t*-tests across conditions (400 trials); *n* = 5 subjects.

In five subjects, we tested two variants of the HDR white block in which we manipulated the degree of luminance variability in the 12 flankers that were nearest in luminance to the target by adjusting them to either the identical luminance as the target (low variability or same, slope 0) or half the original luminance stepping (medium variability or similar, slope 0.5) (Figures 6A and 6B), with the original HDR white condition having high variability (slope 1.0). As in the standard blocks, the positions of the flankers of interest were balanced within each image. Notably, the number of patches and their rules for spatial arrangement were identical between the medium and high luminance variability, and the brightest and darkest luminances remained the same, thus holding constant many factors of concern for lightness theories based on articulation, lightness anchoring, and edge integration (Zemach & Rudd, 2007).

At medium luminance variability, all five subjects had a significant bias toward facilitation in the similar condition (*p* < 0.05 for each subject, Psignifit of 200 trials per condition per subject; population bias 13.9 ± 7.1; *p* = 0.012, unpaired *t*-test; *n* = 5), which was abolished in the brightest condition in all except one subject who had a significant bias toward suppression/assimilation (–3.0 ± 4.8; *p* = not significant; *n* = 5) (Figure 6C). The stronger bias toward facilitation in the similar versus brightest conditions was significant (*p* = 0.015, two-tailed paired *t*-test).

Comparing the similar conditions at medium versus high luminance variability, the magnitude of the facilitation was not significantly different (medium, 13.9 ± 7.1; high, 17.0 ± 8.9; *p* = not significant, paired *t*-test; *n* = 5). This suggests that a medium degree of luminance variability in the similar condition is sufficient to induce facilitation. In the brightest condition, the difference was significant (medium, –3.0 ± 4.8; high, 16.8 ± 12.2; *p* = 0.012). The fact that the facilitation was abolished, rather than weakened, in the medium variability condition suggests that the 12 patches at medium luminance variability may have anchored the target more strongly to this framework, such that its mixture of 45° and 135° Gabor orientations outweighed the contextual effects from the six brightest flankers.

For low luminance variability, one subject had a significant bias toward facilitation in the similar condition, and two subjects had a significant bias toward suppression in the brightest condition. Across subjects, there was no consistent bias in either the similar or brightest conditions (*p* = not significant for both). These mixed results for low luminance variability are consistent with the mixed results in the uniform block. This is not entirely unexpected, as 12 flankers were identical among low luminance variability, similar condition, and the uniform block, and their subjective experiences were similar.

Overall, the results across low, medium, and high luminance variability indicate that the degree of luminance variability in the surround of the target influences the threshold bias in this task. Furthermore, in the medium variability condition, there was an additional clear relationship between luminance similarity and threshold bias. We surmise that the medium luminance variability invoked stronger grouping by luminance similarity than the high luminance variability condition, in which all patches were evenly log-linearly spaced in luminance. Notably, these results clearly support luminance similarity, not luminance identity, demonstrating a strong contextual effect on facilitation, consistent with the idea of articulation that providing additional gray surfaces near the target luminance may increase their use in computing contextual effects. Our results suggest that articulation matters not only for lightness constancy but also for facilitation, reinforcing the suggestion that strong ties exist between luminance and edge processing in early visual cortex (Hung et al., 2007; Hung et al., 2010).

## Discussion

An ongoing challenge to Army modernization is how to develop autonomous teammates that can function in the real world for effective teamwork. Recent advances in machine vision, based on deep neural networks (DNNs) trained on large SDR photographic and synthetic databases, have resulted in substantial improvements in machine vision capability, but a substantial problem remains of unexpected misclassifications, thus limiting machine vision credibility and requiring too many user interventions to be practicable. For example, a recent report showed that DNNs trained on ImageNet photographs were overly reliant on texture for object classification and were easily fooled by synthetic images in which object surfaces were replaced by other textures (Baker, Lu, Erlikhman, & Kellman, 2018; Geirhos, Rubisch, Michaelis, Bethge, Wichmann, & Brendel, 2018). Whereas biological vision has many mid-level processes to support resilient generalization, such processes are absent in DNNs. This is evidenced by the limited ability of DNNs to explain brain activity in many visual areas and by the ongoing challenge to resolve the disjunction between machine and human patterns of classification errors. A strategy to resolve this capability gap is to incorporate biological resilience into machine vision.

Our investigation of HDR luminance normalization aligns with this strategy by building on biology's advantage in normalizing HDR images, with the further aim of building tone-mapping and saliency models that can feed into DNNs, augmented reality, and cockpit displays to improve overall performance. We took the approach of focusing on contextual processing in V1, which has been well explored for the case of static SDR images and is supported by a large body of work in human and animal behavior, anatomy, and physiology. We advanced it toward real-world application by adding HDR luminance dynamics consistent with gaze shifts in naturalistic scenes. We obtained unexpectedly strong behavioral results showing contextual facilitation following abrupt darkening, even for high-contrast targets, and an unexpected phenomenon of contextual luminance-similarity-dependent facilitation that is consistent with traditional Gestalt theories of grouping. Both of these results challenge models based solely on divisive or subtractive normalization and recurrent excitation and inhibition that would predict stronger effects from the brightest flankers (Carandini & Heeger, 2012; Chen & Tyler, 2001; Chen & Tyler, 2002; Foley, 2019; Li, 1998; Li, 2011).

The results cannot be explained by factors such as contrast-dependent sensitivity of neurons to surround stimuli (Sceniak, Ringach, Hawken, & Shapley, 1999) or retinogeniculate adaptation effects, because these factors were balanced across the brightest and similar conditions. One possibility is that, because the display used in these experiments was considerably more complicated than those in classic flanker studies, including temporal luminance transients and patch edges that do not exist in traditional flanker displays, the spatial frequency energy of the patch edges may have contributed to these effects. Notably, some contemporary theories of lightness (edge integration theories) posit that edges, or more precisely, spatially directed luminance change, may be the signal that the brain uses to compute lightness percepts (Rudd, 2017). Our results are consistent with and expand upon edge integration theories and cannot be explained solely by mechanisms based on divisive normalization.

The results open a new direction for modeling, supported by reports of luminance representation in V1 neurons (Kremkow et al., 2016; Yeh, Xing, & Shapley, 2009), that contextual effects are supported by luminance-specific processes. Our future work will be to incorporate these results into computational modeling, based on modeling of luminance, recurrent excitation/inhibition, and feedback processes and on HDR saliency.

These results highlight a limitation of standard laboratory displays for studying biological vision—namely, the limited generalizability to real-world luminance dynamics. By simply widening the luminance range and adding an abrupt darkening, we observed novel phenomena (facilitation to high-contrast targets, flanker effects driven by luminance similarity to the target, and dependence on degree of luminance variability) that should be useful for bringing models of lightness constancy more in line with real-world vision.

### Variability across subjects

We note that in extreme cases, where the flankers had the same versus merely similar luminance (e.g., low luminance variability and uniform blocks vs. medium luminance variability), the lack of luminance variability produced highly variable results across subjects; that is, the lack of strong articulation in terms of luminance variability may have weakened the anchoring framework needed for facilitation, enabling the emergence of top-down attention or heuristics (Gardner, 2019). Put another way, luminance variability in the surround causes the observer to preferentially compare the target to similar features in the background scene in order to reduce cognitive load, and when that luminance variability is missing individual biases emerge regarding the relationship between target and surround.

Interestingly, individual variability was also high in the high luminance variability condition. We considered that target visibility may be challenging after strong (100×) darkening, and indeed the psychometric curves became shallower and confidence intervals wider (Figures 3A, 3D, 4A, 4D). However, it would not explain why the grouping by luminance similarity was consistent across subjects in the medium luminance variability condition with the same strong 100× darkening. It is more likely that, as in the case of low luminance variability, the evenness of the log-linear steps at high luminance variability weakened the strength of grouping effects (articulation), compared with the medium luminance variability condition, and this was compounded by the strong darkening. Further investigation of luminance adaptation effects, such as considering the time allowed for adaptation, is warranted in future studies.

We also note the consistency of the results at medium and high luminance variability in the similar condition, indicating that these effects were sustained within subjects across an interval of over 1 year, indicating that the core effect is robust to changing heuristics and slow changes in visual experience, and they do not decline with familiarity. The overall consistency and reproducibility of the HDR and SDR facilitation supports the suggestion that these facilitation results are primarily driven by automatic grouping mechanisms in early visual cortex but individual heuristics may emerge under ambiguous conditions when articulation is weaker.

### Generalizability

The generalizability of these results is limited by several factors. First, our test range of 3333-to-1 is still well below the 100,000-to-1 range of some natural scenes. However, by capturing a substantial portion of both mesopic and photopic ranges, our display captured the key transition between indoor and outdoor illumination that poses some of the most frequent HDR luminance challenges. Another limitation is that in our target discrimination task, the subject maintained fixation while the luminance changed, whereas such luminance changes typically occur across gaze shifts in a static scene. An important difference is that, in gaze shifts, the visual system has pre-saccadic information about the luminances at the target post-saccade. It is unknown whether this would alter our behavioral results, and it would be worthwhile to repeat this experiment under controlled free viewing, where the subject shifts his gaze from bright to dark regions in a static image, and to investigate how these effects are modulated by temporal dynamics (Burr & Morrone, 1996). Finally, another factor limiting the generalizability of these results is that optimal machine vision may need to encompass both human capabilities and super-human capabilities. Depending on constraints such as size, weight, and processing power, it may not be optimal from a teaming framework perspective for the machine vision to exactly match biological vision. However, we would argue that in most expected uses of autonomy, where the drone or vehicle is mostly autonomous at the front line, reducing the frequency of required user interventions means that the largest sources of misclassification failure should be rooted out. A common denominator of many failures is improper normalization of the visual input, so tackling this issue while taking advantage of DNN advances for later processing efficiently leverages ongoing advances in biological and machine vision.

## Conclusions

These results advance our capability to model biological vision under real-world luminance and to develop resilient and intuitive real-world machine vision, by discovering contextual HDR luminance mechanisms associated with the primary visual cortex. We showed for the first time that abrupt darkening (as would occur during gaze shifts) induces facilitation for high-contrast targets. Surprisingly, the degree of facilitation depended on the degree of luminance variability in the surround. The effect required an HDR stimulus to be observed and was much weaker (but significant) under SDR conditions. Furthermore, there is an additional clear relationship between luminance similarity and threshold bias. At medium luminance variability (10× darkening, or 100× darkening plus medium luminance variability for 12 patches), the facilitation effect was abolished in the brightest condition but preserved in the similar condition, supporting a role for grouping by luminance similarity and orientation in models of vision. We also showed that by simply adding darkening, the classic case of suppression by uniform flankers became ambiguous, with large individual variations toward both facilitation and suppression, and that this ambiguity extended to uniform flankers embedded among the HDR flankers. These discoveries were enabled by our development of an HDR display research platform with improved characteristics of over 100,000-to-1 contrast ratio and pseudo 11 bits, compared to standard SDR displays that are typically limited to below 1000-to-1 contrast ratio and 8 to 10 bits. Together, these results raise new questions for modeling visual perception of real world scenes, leveraging biology's substantial advantages in handling high-dynamic-range images.
